# Koch’s Method with Tuberculosis

**Published:** 1891-02

**Authors:** James Law

**Affiliations:** Cornell University, Ithaca, N. Y.


					THE JOURNAL
OF
COMPARATIVE MEDICINE AND
VETERINARY ARCHIVES.
Vol. XII. FEBRUARY, 1891. No. 2.
KOCHS METHOD WITH TUBERCULOSIS AND THE
EARLIER USE OF CORRESPONDING PRODUCTS IN
OTHER CONTAGIOUS DISEASES, READ BEFORE
THE NEW YORK STATE VETERINARY MEDICAL
ASSOCIATION.
By James Law, F. R. C. S., Cornell University, Ithaca, N. Y.
Koch has at last made known the source of his lymph. It
may be called a solution, in a mixture of equal parts of water and
glycerine, of the products of the culture of bacillus tuberculosis,
or of the growth of tubercle. Stated more distinctly still, it is the
poisonous chemical products of the growth of bacillus tuberculosis,
or a part of such products. The fact that it is insoluble in
alcohol suggests that it is more probably a ptomaine or organic alkaloid
of fermentation,than a toxalbumin. To many doubtless the
revelation will hardly be a surprise. The operation of the poison
alike on the active local tubercle and on the tuberculous systems
suggested as a factor the poisons normally acting in tuberculosis,
and the anyalsis by the Parisian chemists greatly strengthened
this suspicion.
Now, however, that surmise is changed to certainty, and
that the active agent in Kochs lymph is known to be the chemical
poison or poisons produced by the bacillus in its vital process,
the question at once arises, have these products ever before been
used'in medicine ? To this the answer is emphatically yes. The
evidence of this I shall give below. Meanwhile let me say that
nature herself displays the process in the great class of germ
diseases in which the microbethe living parasitic cause of the
diseasedoes not live in the blood, but confines its attack to one
circumscribed area, yet confers an immunity from the attacks of
that disease upon the entire system. As instances of this may be
adduced cowpox and lung plague and mild cases of local anthrax.
All the modes of conferring immunity by inoculating weakened
or partially devitalized virus, or single germs of the more potent
virus, or small doses of virus into the blood are in the main methods
of availing of the same principle. The weakened germ and
the single germ alike perish in the seat of inoculation by reason
of their inability to struggle successfully with the more numerous
and more potent tissue nuclei and the migrating cells. The germs
thrown into the circulating blood are in the case of certain diseases
like lung plague, similarly overcome and devoured by the
myriads and myriads of actively moving blood globules, and thus
fail to plant colonies in the tissues and thereby start local disease.
What then is it, which permeating every tissue and entering every
cell and nucleus, confers upon the whole body a subsequent immunity
from the attacks of the inoculated microbe ? Manifestly
the chemical prisons, the products of the microbes life, to which
these tissues and cells become habituated and, insusceptible.
Those who avail of these methods may not. haye recognized the
full value of these chemical poisonous products in the absence of
the living germs, hence they have not felt justified in leaving out
the dangerous germ itself, yet their results are undoubtedly due
to the soluble products which permeate every part of the system,
and not directly to the germ which is confined to the narrow area
of tissue where it was inoculated, or, in the other case, to the circulating
blood into which it was thrown. More than this, as I
hope to show later, the ptomaines and other poisons have been
experimentally used as medicineapart from the living germs
for at least eleven years past, so that the separation of these chemical
poisons from their living bacteridian sources, and their
employment in medicine is not a new thing, nor was the justly
famous Koch the first to use them apart from the living microbes
for this purpose.
The new departure now made by Koch is not, therefore, the
separation of the ptomaines and tox-albumins from the living
microbe for medical use. That had been already accomplished.
It is rather the application of such separated chemical poisons to
the cure of already existing disease. Kochs departure has been
the introduction of these agents into the field of therapeutics
they had already been introduced into prophytactics. The
only apparent exception to this is in the methods of Pasteur for
the protection of the individual bitten by a rabid animal. But
this is no real exception, because, ist, the Pasturean inoculations
for rabies are not made with the chemical products alone, but
with these plus the microbes at first of a low vitality and potency,
and then day by day of a stronger and stronger quality. 2nd, the
Pasturean inoculations for rabies are prophylactic, being intended
to habituate the brain and other nerve centres to the action of the
soluble chemical poisons, while the living microbe is still confined
to the region of the bite, so that such nervous centres shall
have acquired an insusceptibility before the living germ can reach
them to a viable condition. The tardy progress of the microbe of
rabies to the nerve centres is based on the fact that the blood is
highly inimical to the line of this germ.
From the standpoint of this role of the ptomains, &c., I propose
to shortly canvass Kochs method and somfe of its precursors,
and to inquire into a number of the successive steps through
which the science of medicine has advanced to its present state in
this line of prophylaxis, and it must now be added of treatment.
With this in View it may be well to trace some of the leading
landmarks in the gradual discovery of those poisonous products
now known as enzymes and ptomains.. Strangely enough the
belief in chemical poisonous products antedates by many years
the recognition of living germs as the cause of disease. Poisoning
by decomposing meat and fish had long been a familiar
occurrence when early in the eighteenth century Haller experimented
by injecting watery extracts of putrid material into the
veins of animals, and found that death resulted. In 1820 Kerner,
investigating the question of poisonous sausage, came to the
conclusion that the toxic principle was a fatty acid combined with
a volatile principle. Otturs pursued similar investigations, but it
was only in 1856 that Panum positively demonstrated that the
poison was a chemical poison which was soluble in water and
capable of filtration from other and insoluble bodies. He found
in the decomposed flesh of the dog a toxic product, soluble in
water, insoluble in alcohol, non-volatile and indestructible by boiling.
Of this chemical poison 0.012 of a gram proved nearly fatal
to a small dog. Panum, however, went further and succeeded in
isolating another chemical poison from the putrid meat which
was easily separable from the first by the fact that it was soluble
in alcohol. This alcoholic extract injected into a dogs jugular
threw him into a deep sleep for twenty-four hours, after which he
awoke in apparent health. To Panum, therefore, we owe the first
recognition of two classes of products of bacteridian growth,
which act on the animal system as chemical poisons, and which
have now come to be recognized as ptomaines (rtTGopia cadaver),
which are basic or alkaloidal, and toxic. albuminoids of a non"
basic nature.
Following Pasteur came Weber, Hemmer, Schwenninger,
Peace-Jones, Dupre, Zuco, Bergmann, Schmiedeberg and others,
and the true basic or alkaloidal nature of the product which is
precipitated by alcohol was established.
The poisonous properties of the chemical products were after
a long exposure to heat, which must have killed any living germs,
for a time antagonized by the theory of causation by living
germs, and so late as 1879 such an accomplished observer as Dr.
Lewis argues the absence of living germs in septicaemia on the
ground that septicaemic poisoning can be produced by purely
chemical products.
But the permanence of that theory was impossible. Chau-
veau, Bert and Toussaint showed in the case of Anthrax that the
filtration of the virulent liquids through an unglazed earthenware
filterer rendered the filtrate noninfecting, while the solids retained
in the filterer preserved their full potency. They further demonstrated
that an overdose of the filtrate was speedily fatal, killing
the animal in twelve hours, while a dose of the solids left on the
filter did not kill until after thirty hours. Here we had at once
the prompt poisoning by an excessive dose of the chemical poison
and the slower poisoning by the implanting of the anthrax germ,
and the slow production and increase within the system of the
same chemical poisons as the germ grew and multiplied. Koch
had obtained similar results with putrid fluids inoculated on mice.
To those who had been following the course of experimentation
the field was not clear for a further advance, and such further
advance had indeed become inevitable. A multitude of facts and
observations testified that it was to the chemical poisons we must
look for the direct toxic agents, while the microbes acted mainly
as the products of such deleterious chemical products. In many
cases like cowpox the microbe might be confined to a circumscribed
area of tissue, but the the soluble chemical products pervaded
the whole system and rendered every tissue immune from
future attack. In some cases, indeed, as in cattle lung-plague,
the blood had been proved to be fatal to the germ, which thus
remained confined to the part, lung or tail, where it had been
implanted, but in case of survival no tissue in the body would
thereafter furnish a favorable fluid for its inoculation and growth.
It was inevitable, then, that the next step should be taken
that of employing, the devitalized chemical products of the
microbe of a specific disease to render the system proof against
such disease; and it was natural that Toussaint should be the first
to enter the field. Devoted as he was to the line of experiments
at government expense, in a national school of veterinary medicine,
he had satisfied himself that the survivors of an attack of
anthrax did not contract a second attack when exposed or inoculated.
He therefore, in 1880, instituted experiments on sheep by
heating the virulent anthrax fluids to 550 C. for from ten to
fifteen minutes, and with this product inoculating the animals to
be protected. In a short time he had ten animals thus inoculated
that resisted all ordinary inoculations with anthrax virus. Then
a crucial experiment was institued at Alfort, in which twenty
sheep were inoculated with his heated anthrax liquids, and unfortunately
four of the number died of anthrax. Sixteen survived,
and thereafter proved insusceptible to anthrax. The partially
unfortunate result and Toussaints increasing ill-health would
appear to have deterred him from further advance in the same
line, and no other European observer seemed then to appreciate
the value of his results.
Experiments were, however, being made in the same direction
in America. Dr. Salmon was in 1880 experimenting on fowl
cholera, and I was dealing with swine plague, both for the National
Department of Agriculture. Each independently of the
other undertook to test the protective action of the ptomaines
Dr. Salmons observations on the chicken disease led to negative
results, the ptomaines being either volatile or destructible by
heat. In my experiments with swine plague I was more fortunate.
But to show my views at that date and the results I
obtained, I shall quote from the Department of Agriculture Report
on Contagious Diseases of Animals, 1880-1, pp. 135-146:
  Bacteria Intoxication and Bacteria Infection
  In all diseases caused by microphytes there are two associated
but distinct deleterious agents to be taken into account.
 1st., the organism which is introduced from without, and mul-
 tiplies in the body of the patient; 2nd, the chemical products
  elaborated by the growth and increase of the imported organism
at the expense of the vital liquids. The two have been
 aptly named bacteria infection and bacteria intoxication. Each
 may be injurious and soon fatal, yet each has its special mode
of action and its limitations, so that we can estimate with a
 reasonable amount of certainty the probable results in the two
cases.
  In bacteria- infection the self-multiplying organism is intro-
  duced into the body, and if it finds a suitable field for its growth
  it undergoes an indefinite increase, and may undermine the
health or destroy life in one of various ways; for example by accumulating
in the capillaries, arresting the flow of blood and
  abolishing the functions of vital organs, or leading to local
abscess or gangrene ; by obstructing oxygen and other essential
elements from the. blood, and resolving this vital, fluid into a
poisonous in place of a life-giving streamor by reproducing
itself into myraids, elaborating a vast amount of noxious chemi-
  cal products and killing fly poisoning. The bacteria, intoxication
or poisoning, on the other hand, is effected directly by the
 products of the growth of the bacteria,, or in other words, by a
 chemical compound incapable in itself of reproducing or increasing
its substance. The respective powers and limitations of the
 two poisons may thus be mapped out with great clearness.
 It is manifest that from bacteria infection may be derived all
.the evil results; of bacteria intoxication, in addition to certain per-
 nicipus actions. peculiarly its own. The germ being a living
 organism, with limitless powers. of. growth, it is manifest that
 apart from the power of the system tp support it, there can be no
. boun^ to the amount of chemical poisonous product it may gen-
 erate, and thus do its own special .work of destruction of the
 essential constituents of the blood,,, deoxidation of the vital fluid,
  plugging of vessels, local abscess and gangrene, it must ever add
 the poisonous influences of its purely chemical products. But it
has its limitations as well which do not belong to its products.
, In several bacteridian diseases the system will not sustain nor
.nourish the bacteria with the same readiness a second time, if at
 all. The system that has once sustained an attack does not
 readily succumb to the same ^gain. An incomopatibility or an-
 tagonism has been established between the system thus protected
and the bacterian, and henceforth the system may be repeatedly
 inoculated with the bacterian with the most perfect impunity.
This cannot be said of the chemical products of the bacteria
growth. These, like all chemical poisons, will act again and
  again upon the same system with little difference in effect and,
 if a partial tolerance is acquired it can only be to a limited extent
 and after long exposure to their action, as tipplers acquire a toler-
 ance of alcohol, or opium or arsenic eaters of these respective
 poisons. Kill the microphytes with the injecting bacteria liquids
 and the chemical products will act in exact ratio with the dose
  administered, and no amount of experience with the poison will
prevent an excessive dose proving fatal. The action moreover
 will be prompt, and if it does not produce fatal results at an early
stage, it will gradually subside, for since the poison cannot mul-
  tiply itself its effects must steadily decrease with its elimination
 from the system. With bacteria infection on the other hand the
  evil effects must be somewhat delayed to allow of the reproduc-
 tion of the germ and the production of the chemical poison, and
 thus the disorder of the system will undergo a progressive devel-
  opment In another respect we may conceive of bacteria infection
being limited in its evil results. If the bacteria increase slowly,
  the system will be likely to become somewhat habituated to the
 influence of the poison and insusceptible to it, so that by the time
  the disease reaches its height the system may be able to bear with
, impunity a quantity of the poison which it could not have
tolerated had the .same amount been introduced suddenly and
 before the economy had become inured to its influence. 
 In illustration of the separate action of the bacteria and
 their products Kochs experiments on mice with putrid fluids are
 most instructive. * * * Koch injected , putrid liquids under
the skin of the mouse, and found, when the amount used had
 been excessive, that the mouse died in 4 few hours from the
effects of the chemical poison, and that not a bacillus could be
  found in the blood within the vessels. If, on the other hand a
 minimum amount of the putrid liquid was used, as by making a
slight scratch with a lancet, the tip of which had been dipped in
  the liquid, and if the mouse survived the primary danger of death
  by the chemical poison it died in the course of about two days of
  bacteria infection and the blood was found swarming with bac-
 teria. Similarly Chauveau found that Algerian sheep, that are
naturally insusceptible to anthrax and which had 1 successfully
  resisted inoculation with a minimum amount of the virus, fell
 victims to the disease if an excess of the poison were injected
  under the skin, or if a second and third inoculation were practised
before the effects of the first had passed off. Finally Cossar-
 Ewart, and Burdon-Sanderson found that when anthrax liquids
had been devitalized by exposure for some time to compressed
 oxygen(i2 atmospheres)and when the germs had lost their power
 of propagation and increase, the fluid still proved injurious, and
 even fatal to animals on which it was inoculated. With the
4 4 vital germ destroyed these evil effects could only come of the
4 4 remaining chemical poisonous products, which retained their
4 4 original potency.  
4 4 My own experiments with the virus of hog-cholera tend to
4 4 establish the same fact in that disease. When I had subjected
4 4 the virulent fluids for an hour to a temperature oscillating bet-
44 ween 130 andi4o F., and then inoculated them on the pig, I
4 4 found that the reSult was a certain amount of constitutional disorder
and ill health, which did not however, go on to a fatal
issue. * * * Similarly when I injected into the system large
4 4 quantities of the virulent fluids, I found that death took place
4 4 almost without exception, even in animals that had resisted
44 ordinary inoculations. Of this mortality we may find an ex-
4 4 planation in the ebjile state of the system induced by the presence
4 4 of the chemical poisons. * * * It is not the increase in the
4 4 number of the bacteria alone, nor the access of fresh, and there-
4 4 fore more potent, germs, that have the evil effects, for in the in-
4 4 fected system there is practically no limit to the multiplication
44 of the bacteria, and these, in place of being weakened, are often
rendered more potent by passing through a succession of animal
systems. * * *
4 4 Is future protection secured by the action of the chemical pro-
44 ducts alone, or is the presence in the system of the bacteria
4 4 essential ?  
Under this head are discussed the doctrines of immunity by
elimination of systemic products necessary to feed the microbe ; by
the deposition in the tissues of poisons inimical to the microbe,
and of condensation of the lymph channels and glands, the first
two being contradicted by the growth of the bacteria in the bouillon
made from the flesh of an insusceptible animal, and their growth
in a flask charged with their chemical products provided fresh
meat-infusion is introduced. The third theory is disproved by the
fact that different bacteridian diseases all alike resulting in such
condensation of lymph channels are not mutually vicarious of each
other. The doctrine of physiological resistance is shown to agree
with all the facts. and the imunity of the calf whose dam has
passed through the bacteridian disease in the latter peroid of the
gestation is especially dwelt on as evidence.
44 This view is further strengthened by the fact that though
an animal, that has acquired an immunity from a specific disease,
4 4 afterward produces offspring which are susceptible to the disease
41 we question, yet it has been shown in the case of anthrax, that
 if such immunity on the part of the parent has been acquired by
  a non-fatal attack of the affection during advanced pregnancy,
preservative effect is extended to the foetus as well. Here the
 foetus has advanced beyond the condition of an ovum, or of simple
  embryonic cells or tissues and is already well formed, with all
its differential bones, muscles, tendons, brain, nerves, vessels
 and viscera. The nuclei presiding over the growth of these
  different structures are henceforth fixed in their powers, and any
  habitude impressed upon them may now be permanently pre-
 served just as it is in the adult animal.
This consideration serves to fortify the doctrine that the
 immunity from a contagious disease acquired by a first attack is
1 due to a habit, or acquired power of endurance or resistance on
 the part of the living cells or nuclei of the animal body. This
 better accords with and explains observed facts, and is liable to
fewer objections than any theory of the subject that has come
 under our notice. * *
 To return to our question, Do the observed facts accord best
 with the idea that protection is acquired by the action of the
 chemical products only of the bacteria, or is the presence in the
 system of the bacteria essential? As the question appears to
  us every thing serves to support the first conclusion. *  * *
The facts attending the acquired immunity of the advanced but
 unborn offspring of an anthrax mother seems to be almost con-
 elusive on this question. The blood of the dam (cow) may be
  swarming with bacteria, but these have never been found in the
 blood of the foetus. It is only reasonable to conclude that they
  have never entered the body of the foetus, or if otherwise, that
  they have perished very soon after they entered. The chemical
 products on the other hand, being soluble in the vital fluids pre-
 sumably enter the foetal system along with the natural secretions.
The offspring when born proves refractory to the anthrax, so
  that there is the strongest presumption that it has been fortified
 by the action of the chemical products of the anthrax upon its
system before birth. In this case immunity cannot well have
 resulted from any action of the growing and multiplying bacteria
on the blood or living tissues, for the evidence is all opposed to
 the idea of their presence, at any time, in the foetal system, much
more to their growth and propagation there. Yet here unques-
 tionally the disease in the mother has produced an insuscepti-
bility in the foetus, such as would occur had it been itself the
 subject of the disease. It follows almost of necessity that the
  introduction into the system of the chemical products of the
 bacteria is equivalent in a protective sense to the introduction of
 the bacteria themselves. But the pure chemical products cannot
  undergo increase in the system ; therefore we can graduate the
 dose of these as safely as we can a dose of opium or rhubarb.
With this presumptive evidence we are prepared to study
the direct results of the introduction into the system of the chemi-
cal products of anthrax, and swineplague, made with a view of
securing an insusceptibility to the respective diseases in the
 future.
Here follows record of Toussaints experiments with anthrax
which need not be repeated.
 My Results" With Swineplague.
 A pig was injected with one drachm of virulent swine-plague
blood which had been repeatedly heated to 130, 150 and 200
 F., and a month later with an equal amount of virulent blood
which had been raised to 130 F., for thirty minutes, and the
 day following for three hours. This caused some loss of appetite
 and appearance of ill health, but no very appreciable fever.
  Thirteen days after the last operation this pig was placed in a
small pen with a pig suffering from swineplague,and at intervals
 of a month was twice inoculated with the virus of swine-plague,
  but all without evil consequence.  
Another pig was injected with a drachm of the mucous-
 covered faeces of a pig suffering from swine plague, the infusion
  having first been filtered and heated for one-half an hour to
 130 F. until all movement of the contained bacteria had ceased.
  As in the other case there was some evidence of ill health but
no material fever, and on the thirty-eighth day the subject was
  placed in a small pen with a sick pig.-i Afterwards at intervals
 of a month ; it was twice innoculated with' (swine plague) virus
but successfully resisted, and maintained good general health.
 A third pig was injected with a drachm of pork infusion
  which had swarmed with bacteria resulting from an inoculation
*  with infusion of putrid maize. Before inoculating it on the pig
 the pork infusion was heated to 140 F. for three hours in succession.
There resulted some derangement of health, slight
fever, and a local swelling in the seat of injection. When this
 had subsided, on the fourteenth day the pig was placed in a
 small pen in company with a diseased one. Nine days after
4  she had a sharp attack of swine plague, which lasted eighteen
4days and led to much loss of condition. Later, at intervals of
44 one month, she was twice inoculated with active virus of swine
44 plague, but on each occasion without any further ill result.
44 On the last occasion of the inoculation of these three pigs
44 a fresh pig was inoculated with the same virulent matter, which
44 caused considerable fever with a temperature varying from 104
44 to 1060 F., but from which the subject finally recovered.
4 4 Here, then, we have two pigs protected from the noxious
4 4 action of the swine plague virus, by being first brought under
44 the influence of the chemical products resulting from the growth
4 4 of this virus in the system. We have, further, another pig
4 4 treated in the same way with the products of an ordinary
4  putrefactive fermentation in a pork infusion, which had been
4  similarly devitalized by heat, but this fails to secure the same
4 4 immunity, and this pig suffers surely from swine plague when
4 4 made to cohabit with a victim of that disease. Later this pig
4 4 and the two others successfully resist two successive inocula-
44 tions with swine plague virus, while a fourth pig inoculated
4 4 with this same virus sustains a considerable, but not a fatal
44 attack.
4 4 The experiments, it is true, are limited in number and
 liable to the objection that the results may have been accidental
4 4 coincidences, yet so far as they go, they support the theory that
44 the chemical product of the swine plague germ, when deprived
4 4 of its living microphytes affects the system so as to render it, for
4 4 the future, insusceptible to the attacks of such germs. When
4 4 in connection with the fact that swine plague rarely recurs in
4 4 the same individual, that, as in the case of other diseases that
4 4 attack the same animal but once, the most rational explanation
44 is that it is the deleterious products of the disease-germ, and not
4 4 the germ itself that affects the system so as to secure this im-
4 4 munity, and finally considering that in the closely allied disease
4 4 of anthrax Toussaint has secured a similar insusceptibility
4 4 by an identical process, it is altogether reasonable to suppose
44 that we are here furnished with a system of prevention which, if
4 4 carried into general practice, would reduce our present losses
4 4 from swine plague to a comparatively insignifiicant figure.  
The report goes on to show the danger of popularizing this
method since all the fatal diseases of hogs vulgarly, but mis-
\ takably known as hog cholera, would be resorted to for the
protective ptomains with unsatisfactory results. Also that protection
from, all these varied fatal swine diseases would be vainly
sought by the use of the ptomains of swine plague. Then follow
summaries of needful precautions, and of the manifest advantages
and disadvantages of the method.
precautions To be observed.
  ist. See that it is the genuine swine plague that is being
  dealt with. This is equally necessary as to the disease to be
  promoted and as to the virus which is to be devitalized for
 preventive inoculation.
2d. The virulent fluid to be devitalized maybe the blood
 of a diseased animal, or the liquid exudation into a diseased
 organ, including the lumen of the bowel. In such cases it is
  best taken at the height of the disease rather than from a
  partially convalescent animal in which the virus may have dis-
  appeared and the structural changes only may have benefit. If
  from a cultivation in pork infusion that should have been pre-
  pared with all due precaution against the introduction of air
 bacteria and with access to air, but which air should not much
 exceed one-fifth of its bulk.
  3d. In exposing this fluid to heat, that should be carried to
 140 F. and retained at that temperature for an hour or more
until, in short, all indication of life in the contained mycro-
 phytes have ceased.
4th. Swine to be operated on must be kept apart from all
diseased hogs and infected places and objects, for with the
 presence of the living germ in the system the injection of the
  devitalized chemical will only tend to aggravate the attack.
 For the same reason all inoculated animals showing symptoms
 of a severe attack and presumably suffering from bacteridian
  infection, in place of the single intoxication with the chemical
  products, should be at once removed from the herd operated
 on.
  5th. In inoculating the devitalized chemical products, the
injection of a small quantity at a time, and its repetition at
  intervals of three days or a week promises to be safer and more
effectual than one large injection. The injection of 10 to 20
  drops at a time and its repetition once or twice, would probably
  secure a greater immunity with less loss of condition and pro-
 gress than if a larger amount were introduced at once.
6th. The animals operated oil should be carefully guarded
 against, infection for three weeks after the last injection of the
 devitalized virus. The presence of the chemical poison in the
  blood and the attendant constitutional disturbance invites rather
than debars the growth of the plague-germ; how the latter
 must be excluded until the former has been entirely eliminated.
 For the same reason the free use of disinfectants in the operat-
  ing yards and buildings will be of the utmost value. So will
  every conceivable precaution against the introduction of disease-
 germs through accidental channels as by other animals, by the
 pork stolen by dogs, as carried by men, &c.
Advantages Promised by this Method.
 ist. It offers immunity from a fatal disease by a method
which does not entail the propagation of the living germ in the
 system of the animal, to be protected.
 2d. It avoids the risk of the preservation, amplification, dif-
 fusion or increase of potency of the disease-germ, all of which
  contingencies are possible in inoculations with a mitigated
 virus.
  3d. It does away with the necessity for an exhaustive disin-
  fection after the animals have been inoculated and have recovered
from its results.
4th. The dose of the devitalized chemical products can be
  so graduated to the strength of the animal that there will be no
4 4 risk of a fatal result When even the mitigated living germ is
4 4 introduced there can no longer be any certainty that it will not
 reproduce itself to a dangerous extent, or that owing to the
4 4 special condition of the system or of its surrounding it may not
4 4 suddenly assume its fatal type ; but with the devitalized chemi-
 cal products we can graduate the dose so as to secure as great a
certainty as in the case of a dose of castor oil or Epsom salts.
 5th. The system can be habituated to the poison and forti-
 fied against it by a succession of small doses, no one of which is
44 at all dangerous in itself, whereas if a living germ were once in-
 troduced, though of mitigated power, it may increase so as to
 develop a power that is altogether unexpected.
 Disadvantages and Drawbacks.
These are few apart from the certainty that, if largely re-
44 sorted to, it will be misapplied by many, to other diseases than
the genuine swineplague, and will thus fall into disrepute.
44 It can do no good, but only harm to animals that are already
44 injected, as it can only add to the deterious products with which
the germ is charging the system.
Its effect can only be evil if the subjects are allowed to
  become infected before the chemical products have had time to
fully affect the system, and to have become eliminated. If this
 is neglected, and early infection is allowed it can only add to the
mortality.
  There is the additional disadvantage that to secure the pro-
 tective products, the production of a virulent germ must be kept
 up, either in the bodies of a successive series of diseased pigs or
in an infusion of pork. The slightest carelessness with regard to
the seclusion of these fluids of poison, or as to the disposal of
their products, may easily become the occasion of a spread of
the worst type of the plague among unprotected animals.
I have quoted thus at length to show that at that date it was
with me no mere chance suggestion but a settled conviction,,
based on my own successful experiments and the long series of experiments
by others which had led up unmistakably to this conclusion.
And this at a time when Koch, whose experiments on
mice had directly indicated the principle availed of, had not yet
apparently grasped the true significance of the plomaines in conferring
immunity and when Toussaint, who was the first to apply
the principle had been nonplused by the unlooked-for results of the
Alfort experiment, and led to conclude that his former successes,
depended on a retained vitality in the virus employed.
In the Department of Agriculture Report for 1882 Dr. Salmon
again records his lack of success with this method in chicken
cholera, and while accepting the principle that immunity is naturally
secured through a power of resistance acquired by the living
tissues by continuous exposure to the action of the poisonous.
Ptomaines, he specifically denies the possibibity of any benefit
from the use of small doses from the latter. On page 310 of the
Dept. Report he says :   That this method was entirely inefficient
 however, was completely demonstrated, and the conclusions of
 Toussaint and others shown to defend on wrong interpretations
 of the facts observed.
Dr. Salmons Success with Swineplague.
Dr. Salmon seems to have returned to his method in December
1885. In a paper read before the Biological Society of
Washington, Feb. 20, 1886, and contributed by Drs. Salmon and
Smith, these gentlemen record their experiments on six pigeons,
with swineplague virus sterilized by heat (58C. for ten minutes).
One pigeon No. 8, was injected with 0.8 cc. of the heated culture
fluid and 51 days latter it was inoculated with 0.75 cc. of strong
swineplague virus with the result that it died in 48 hours. A
second pigeon No. 10, was injected with 0.4 cc. of the heated virus,
and on three subsequent occasions at intervals of 28, 8 and 8 days
it had an inoculation of 1.5 cc. of similar heated virus, and on the
seventh day succeeding the last injection it had an injection of
o. 75 cc. of strong virus, but remained well. Two other pigeons
had injections of 1.5 cc. of the heated virus on three occasions with
intervals of eight days and a third had two injections of the same
amount with an interval of sixteen days, and seven days after the
last each was injected with 0.75 cc. of strong virus but all remained
in good health. Finally a chick pigeon which had not been injected
with the heated virus was the same day with the five others
injected with the same amount (0.75 cc.) of strong virus and died
in 24 hours. They thus sum their conclusions :
 1 st. Immunity is the result of the exposure of the bioplasm
  of the animal body to the chemical products of the growth of the
specific microbes which constitute the virus of contagious
fevers.
2d. These particular chemical products are produced by the
1 growth of the microbes in suitable culture fluids in the labora-
 tory as well as in the liquids and tissues of the body.
 3d. Immunity may be produced by introducing into the
*  animal body such chemical products that haye been produced
in the laboratory.
This paper has been generally accepted as crediting its
authors with the discovery of this principle of immunity by
chemical products of the life of the germ, a principle which I set
forth so extensively in its bearing on the same disease five years,
before. It is true that I did not experiment with sterilized laboratory
cultures, but assumed as self-evident that the disease germs,
grown in a suitable medium and with a limited supply of air
would elaborate the same poisonous products.
EXPERIMENTS ON PIGS BY DRS. SALMON AND SMITH.
In the Report on Hog Cholera issued by the Bureau of
Animal Industry in 1889, Drs. Salmon and Smith record the
result of injections with sterilized virus on twenty-two pigs,
which, at first sight is anything but reassuring as to the value of
such products in protecting these animals against the disease.
Of the whole number treated with the sterilized virus twenty died
and only two resisted the disease when subsequently exposed.
The following gives the results in tabular form :
Expo
sure to
Inter- Total Infect-
Num- vals be- sterjHz- 1?n a!ter Mode Did Interval
Exper- berof tween y/J?- last In- JrLa d not Exponent.
Ani- 83z such In-d Xlrf" ocula- Bled' Sick- sure to
mats. fJvVr^ ocula- tionwith sure- en. Death,
ed Virus. tiong> each ng. sterili_
zed
____________________________________Virus._________________________
Days. Days. Days.
Fed Dis
eased In-
lst 2 2 8 18cc. 10 testinesof 2 10-17
Pig put in
Infected
Pen.
Put in
(3 2 3 19CC. 15 Infected 3 31,98, 113
2d { Pen.
(2 0 0 0 2 14. 19
( 2 2 5 20CC. 22  2 29,83
3d 11 2 5 . 20CC. 57  1 34
( 2 2 5 20CC. 22  2
f 2 4 2,2,2 33.5CC. 8  2 15,19
j 2 3 2,2 25.56C. 10  2 13, 15
] 2 2 2 18CC. 8 " 2 13, 15
13 0 0 0  3 13, 19,39
fs ,2 3 40CC. 4  5 I**. 18
Bth 1 1 2 3 33CC. 4  1 18*
11 0 0 01 18
There are some points to be noted in explanation of the
terrible mortality :
1st. The excess of the sterilized virus used, from 18 cc. in two
doses in eight days to 40CC. in two doses in three days. Because
a pig suffering from hog cholera has a great excess of these
poisons in its system it does not follow that that amount is essential
to secure immunity. The single vaccine vesicle protects the
average system. Further, in a disease which, like hog cholera,
produces congestion and even necrotic ulceration of such vital
organs as the intestines and mesenteric lymphatic glands, the
ptomains are likely to endanger lesions of these parts that may
prove lasting, and the embryonic tissue in such lesions would be
especially open to colonization by the microbe. We see an example
of this in the predisposition to tuberculosis where there is
any inflammation of the air passage. It is notorious that after
passing through hog-cholera a pig rarely thrives. It is desirable
therefore to use small doses only of this chemical poison for the
pig and to avoid if possible the production of lesions which would
virtually predispose to the disease.
2d. In the more fatal cases the exposure to infection was
made so early as to forbid the idea that the system had had time
to have completely rid itself of the poisonous chemical products,
and much less to have risen over their profoundly depressing effects.
The intervals of four, eight and ten days respectively between the
injection of large doses of the chemical poisons and exposure to infection
suggests the presence of that toxic effect of the ptomaines
which lays the system open to the microphytes. One hardly
wonders that these subjects died in periods varying from thirteen
to nineteen days thereafter. It is noticeable that the two pigs that
escaped the infection, and the six that survived 29, 31, 34, 83, 98,
and 113 days respectively, were those that had at once had a small
amount of the sterilized virus 19 cc. and 20 cc. and that had exposure
to infection delayed longer (15, 22 and 57 days) after such
injections had been made. It may be that immunity by this means
is not safely attainable on a large scale, yet even this dreadful
table bears testimony to the operation of the principle of protection
by chemical products of the germ, however unfavorable the conditions.
The energy of the disease was abated and the acute disease
was exchanged for the sub-acute and chronic form in all cases
in which the sterilized lymph was moderate in amount and the
subsequent exposure to infection delayed.
3d. It is to be noted that in the case of these experimental
pigs the test was an unusually severe one. The ingestion of the
diseased bowels ten days after the pigs had been injected with the
prostrating ptomaines, and followed by exposure in an infected
pen was a test of extraordinary severity. Again in the other
cases the reporters say that the infection in the pens was unusually
intense, the experimental pigs being constantly surrounded by the
sick and dying, at one time fifteen pigs dying in three weeks, and
at another pigs died almost every day  in the infected pen.
Taken all in all these experiments sustain the doctrine of protection
by the ptomaines although they do not show a large
percentage of survivors.
Experiments with Lung-Plague of Cattle.
Inoculations With Sequestrum.
My next essay in this field was with lung-plague, and my first
experiments were made by diluting and inoculating the juice
squeezed from a sequestrum from the lung of a cow which had
been attacked at Orange, N. J., August 27, 1881. The cow was
killed January 3d, 1882, and January 5th inoculated three yearlings
by injecting forty drops into each lung of each animal using a
hypodermic syringe with long nozzle, which was passed through
the intercostal spaces. Two of the yearlings coughed a few times
but all of them at once took to eating heartily. One, which may
be called No. i, was suffering from a chronic bronchial catarrh,
and one gram of the sequestrum was inserted into a subcutaneous
pouch in front of its shoulder. At the time of inoculation all had
a temperature of 103 F. For two days there was a very circumscribed
crepitation in the seat of the inoculation, before the third
or fourth day this had disappeared in all alike. The temperature
of No. 1 remained at 103 F., that of Nos. 2 and 3 went down to
102 F.
All remained without further change for six weeks and on
Feb. 16, I again inoculated each in the right lung with forty drops
of the slightly diluted juice of a sequestrum taken Feb. 15, from a
New York City cow by Dr. James D. Hopkins. This cow had
been sick eleven weeks before her slaughter. Seventeen days
later I found the temperature of 2 and 3 had risen to 103.50 F.,
and this it did occasionally for eight days but there was no other
indication of illness.
March 16 I inoculated these three and another (No. 4.) in
the right lung with the freshly exuded lymph from the lung of a
cow which had sickened March 13, and was killed March 14.
March 23, No, 4 coughed while temperature was being taken and
this was repeated daily until the 26th when there was slight
crepitus in the right lung. Temperature 103.75 F., pulse *60,
breathing quiet. Temperature on the 25th had been 103.50 F.
Next day her temperature was 102, but on March 29th it was
103.50 and by April 1, and 2 had reached 105.750 F. There was
slight dulness over most of the right lung and swelling of the right
side of the chest. It ate and ruminated but was losing flesh.
(Nos. 2 and 3 showed no reaction.)
Necropsy of Check Case No. 4.
This subject was killed April 22, and showed on necropsy the
right side engorged from shoulder to flank with the usual straw-
colored lung-plague exudate, the pale yellow muscles and bands
of connective tissues being pushed apart so as to give the tissues a
honeycombed appearance, excepting in the central part around
the seat of inoculation where they were condensed and fibrous,
like an organizing false membrane. The right plural sac con-
taihed a considerable liquid exudate and false membranes covered
the lower portion of the pleura and pericardium. The lung was
normal, the inoculating nozzle having manifestly failed to enter it.
The subdorsal lymphatic glands were enlarged, gorged with
blood, and showed a granular appearance.
Necropsy No. i.
On April 13, I had already made a necropsy of No. 1, but
found only the -lesions of the original bronchial catarrh and two
small curdy-looking massesmanifestly tuburclein front of the
left leaflet of the diaphragm and in the spleen respectively.
Inoculations with Sterilized Exudate from Recent Lung-plague.
March 22, I inoculated two yearlings (Nos. 5 and 6) in the
tail with a drachm each of the exudate taken from the lung of a
recent case of lung-plague, and heated for some time to 180 F.
There insued a slight swelling in each in the seat of inoculation
but no general reaction. Thirteen days later (April 4,) these
were tied, one on each side of the sick check case, No. 4, and kept
there until the latter was slaughtered, April 22. April 9 to 10,
the temperature of both rose to 103, 104 and in the case of No.
5, on the 14th and 15th to 105 and 106 then subsided to the
normal (102). There was no indication of any lung disease.
April 21, I inoculated a yearling heifer, No. 7, in the tailand
left flank with the sterilized lymph from the flank of No. 4, killed
same day. This lymph had been kept for hours at 140 to 150
F. There was no local nor general reaction.
April 27, inoculated No. 7 and another yearling, No. 8, with
sterilized lymph that had been heated to 140 for several hours.
There was no reaction.
April 22, I inoculated a yearling in the left lung and in the
trachea with fresh lymph from No. 4, just killed. In all 1% oz.,
of the liquid was employed. From April 24, to 30, the temperature
rangedin the main 104 F. and 105 F. and thereafter to May 20.
oscilated between 104 F. and the normal. Swellings'appeared in the
neck and left side in the seats of inoculation, pulse and breathing
ranged high but no distinct lung symptoms were observed. The
local swellings subsided to two hard masses like hickory nuts and
the general disorder disappeared. Later at the post mortem examination
this animal showed a small sequestrum in the left lung
and old false membranes on the pleura.
Exposure of the A bove to Infection.
On July 15, I sent three of the survivors to Christopher Slade,
Whitehall, Baltimore Co., Md., to be kept in his infected stables
and three to Mr. W. W. Nabbs, Newtown, Queens Co,, I/. I., to
be placed with his infected herd in the close buildings of a town
dairy. Three months later I heard from both gentlemen that not
one of these cattle had shown a sign of lung plague.
Table Showing Lung plague in Inoculations.
Inoculations.
 With Inter- Inocula- Time from
No. of With Old Lymph of va^s ^e" ticms with Cohabited last Ster-
Sub- sflniiaqtnim Amita Tmnff tween Lymph of with Cases ilized In- Result, jects. of Lung Plague Stei? Incula- Acute of Lung j ection to
Plague ilized by tions. Lung . Plague. Exposure
Heat. Plague- to Disease.
Times.' Times. Days. Times. 3 Months. Days.
2 2 42 1 28 Nil.
1 2 42 1 28
1 1 Lung
Plagua
,2 .1 18 Days. 13 Nil.
1 2 5 3 Months. 79 
1 1 3 Months. 79
1 1 Lung
Plague
Further Experiments With Lung plague.
In August 1883,1 inoculated with lung plague exudate sterilized
by heat, a number of cows going into dairy stables in New
York and Brooklyn. One went into an infected stable in 70th St.,
and 2nd Ave., New York, and two to McDonalds, Greenpoint,
which was habitually infected. The dealer P. McCabe, assured
me a year later, that none of these contracted lung-plague.
April 3, 1884, I inoculated with similar sterilized exudate,
eight cows for J. Colman, five for Mr. Reilly, and nineteen for
Mrs. Lynch all in 89th St., New York, seventeen for D. F. Murphy,
92d St, and two for Mrs. Barry, 95th St., and from the later
reports of these parties I have reason to believe that all the cows
operated on escaped the disease which habitually existed in the
locality.
Twice I have had untoward results, in herds wherein the
plague existed at the time. In one case the diseased lung used to
furnish the lymph was an unsatisfactory specimen having little
exudate into its substance and the disease continued to occur in
the herd. In the second case the only lung available had through

out advanced into a state of firm dry-granular hepatization and
had a mawkish heavy odor, and no theromometer could be found
marking above 120 F. As a result the heat employed failed to
sterilized, and the tails swelled as under an inoculation with the
unaltered virus. The result was protective, but many lost their
tails, and one Jersey died in a herd of fifty head. Most of these
cases were reported to the Fourth International Veterinary Congress,
at Brussels, in September 1883. (See Comptes Rendus of
this Congress, pp. 496 to 501.)
Experiments with Anthrax.
Mr. Shepherd, Skaneatles, N. Y., had nineteen head of cattle
attacked with Splenic Apoplexy, January 1883. In a few days he
lost seven adult cattle and one calf, together with a mare and colt
and one pig. Eleven cattle recovered. In March 1884, he lost one
heifer, in May three young calves, in June one mare, July 3, one
horse and July 6, one yearling colt. The cattle first attacked
grazed in a pasture in which was a swamp and where there had
formerly been a slaughter house. The horses in 1884 were kept in
an orchard into which drained the yard used by the infected cattle
in 1883.
July 8, I visited the herd obtained the requisite blood from a
victim of the disease, heated it for an hour to 150 F., and injected
twelve cattle with one drachm each of the sterilized product. One
heifer remained, and by desire of the owner this was left as a test
case. Within two days this thirteenth animal died of anthrax and
the other twelve survived without illness.
In 1885 I operated in the same way on a large herd of cattle
in Columbia Co., N. Y., among which anthrax had appeared. This
herd escaped without a single loss.
From the early eruption of symptoms and the rapid progress
of anthrax it is especially favorable to such treatment in infected
herds. The truly sick can easily be picked out, and the inoculation
of the others is likely to prove protective. The shortness of
the incubation, and above all the absence of occult and chronic
forms of the disease saves the operator largely from the dangers
that beset him in such affections as lung-plague and hog cholera.
Experiments with Rabies.
1st. April 2, 1886, I injected hypodermically in the back of a
rabbit (No. 1) ten drops of a lacteseent fluid made with the brain of
a mad dog who had died of hydrophobia March 30. May 18,
the same rabbit was again injected hypodermically with brain
matter of a dog which died May 17 of rabies. January 14,
(seventy-two days after the first inoculation) this rabbit was found
paralyzed, the paralysis increasing the two following days, so it
was killed June 16, and utilized for further inoculations.
2d. April 3, I injected on the left cerebral hemisphere of rabbit
No. 2 the same fluid (from the human brain) used on No. 1. Rabbit
was dull for three days, but ate its food. After this it was
well and lively up to the 18th inclusive. April 19, in the morning
it was paraplyic but still fed. April 21, the paralysis had be-
come complete, and body was becoming cold. At 4 p. m. it was
found dead. The attack came on the 16th day after inoculation,
the regulation time for rabies resulting from inoculation on the
brain.
3d. April 3, I injected a black and tan terrier No. 3, in the
flank with twenty drops of the same liquid used for Nos. 1 and 2,
but which had been first heated for an hour to a temperature ranging
from 150 to 1800 F. April 19 and 20, two other inoculations
were made in the flank with the same sterilized liquid as before.
April 22, an inoculation was made on the cerebrum of a milky
fluid made by mixing with boiled water a portion of the medulla
oblongata of rabbit No. 2, which died the day before.
May 17, the dog which had lost appetite somewhat for several
days snapped at a stick and later at my leg. It was found' dead
next morning. It is noticeable that twenty-five days elapsed before
symptoms appeared. The rule is that after inoculation on the
brain symptom set in in sixteen days ; it is therefore reasonable
to infer that the first inoculation with sterilized virus had conferred
a partial insusceptibility.
4th. April 22, I inoculated in the back hypodermically a
rabbit, No. 4, with twenty drops of the mixture of the medulla of
rabbit No. 2, diluted with boiled water. May 18, the same rabbit
was again inoculated with brain matter of the dog No. 3. This
rabbit was found dead November 29.
5th. April 22, I added three grains of medulla No. 2 to six
drachms water and heated for an hour to 150 to 170 F., leaving
the vessel wrapped in a rug so that it remained warm till the morning.
April 23, heated the mixture till it began to simmer, superheated
the neck of the flask, and injected one drachm
hypodermically in the back of rabbit No. 5. May 18, inoculated
this rabbit in the back with brain matter of dog No. 3. February
24, 1887, injected on the left cerebral hemisphere ten drops of a
mixture of a healthy rats brain and boiled water. April 15, this
rabbit was in good health when I had to leave home for Chicago.
In view of the claim that healthy brain matter inoculated on
the cerebrum would kill in sixteen days by paralysis, on February
24, 1887, I inoculated the brains of three additional rabbits with
matter from the brain of a rat just killed, but all remained well
when I was called by the United States Government to go to
Chicago April 15.
We have thus traced some landmarks of the incipiency and
development of the doctrine of immunity by the use of the purely
chemical products of the plague-germ. We have seen the principle
applied in practice to anthrax by Toussaint in 1880. We have
seen it applied to swineplague by Law almost at the same date,
and published in the Agricultural Report for 1880. We have seen
it applied to lungplague byAawin 1882 and recorded in the report
of the International Veterinary Congress of 1883. We have seen
it again applied to Anthrax by Law in 1883. We have seen it
applied to Swineplague in pigeons by Salmon and Smith in 1886
and set forth in their paper presented to the Biological Society
February 20, 1886, On a New Method of Producing Immunity
from Contagious Diseases. Finally we have seen the experiments
on Rabies by Law in 1886.
Contrary therefore to the impression that Salmon and Smith
inaugurated this method, the first successful step had been taken
six years before by Toussaint, and the indisputable printed records
show that I had a gratifying measure of success with three plagues
five, four and three years respectively before the successful experiments
of Salmon and Smith on which they based their New
Method. I say this in no disparaging spirit^ as no one is more
ready than I to acknowledge the great work that Drs. Salmon
and Smith are accomplishing for the country. They can well
afford to allow the justice of my claim of priority in this matter.
Obscurities and Difficulties in .Kochs Method.
While the basis of Kochs fluid is the chemical products of
the bacillus tuberculosis the use to which he puts it of a curative
rather than a prophylactic agent introduces an element of difficulty.
The tendency of tuberculosis is to death of the tubercle. The
chemical poisons may therefore be looked upon as necrosing
ptomaines. It would seem therefore as if its sole benefit must result
from the expediting of this process. In a lupus or other
superficial tuberculous product this may prove curative, but in the
case of a tubercle imbedded in the depth ofa solid organ, it puzzles
one to see how the dead mass is to be eliminated and a recovery
secured without a general diffusion of the contained bacilli. Koch
himself allows that the bacilli are not all killed. And now Virchow
on the strength of twenty-one post mortem examinations of individuals
treated by Kochs method asserts that it endangers the
diffusion of the germ and the onset of a general tuberculosis. It
now appears that even some cases of lupus are refractory.
Similarly it would seem as if the method would entail great
risk in case of tuberculosis of the bowel. Here the resulting
necrosis must endanger perforation of the intestine and septic
peritonitis.
But again tuberculosis can .hardly be classed among nonrecurring
diseases. Though its subject acquires in him a partial
tobrance of the poison, yet this does not forbid a continuance of
the tuberculous process for a long lifetime in man and beast alike.
A temporarily dormant state of the disease does not hinder its
breaking out anew in an acute and granalized form under unwholesome
conditions of life. How often do we see one or two
old calcified tubercles, in a system the subject of acute tuberculosis.
Prima facia, therefore, it would seem as if tuberculosis did
not belong to that class of diseases for which a personal immunity
of a reliable kind could be acquired. But if so, is not the benefit
from Kochs method to be limited in the main to the process of
local necrosis of superficial tubercle, and of such deeper formations
as can be safely reached and removed by surgical means ? It
seems that the great bacteriologist himself is aware of this and is
calling in the aid of Bergmann for the subsequent resection of the
diseased joints. If the bacillus escapes from the incrosed tubercle
into the adjacent living tissue the vitality and antagonism of
which is reduced by contact with the ptomaims there is everything
to favor formation of a new colony.
Even the alleged value of the injection as a means of diagnosis
is now falling into- doubt. The fibrile reaction sometimes
occurs in the non-tuberculous, and some unquestionally tuberculous
Subjects do not show it. Koch justly claims a diagnostic value
for the local reaction only. This may be itself incapable of diagnosis
in internal tubercle.
On the whole there seems to be good reason for moderating
very materially the extravagant expectations with which Kochs
tuberculosis treatment was heralded. Much of the unreasonable
claims made for him was doubtless due to the high reputation of
the author of the method, and his known habits of carefulness and
thoroughness, but in this case his perhaps too precipitate launching
of the fact of his discovery, and the mystery with which he
surrounded its real nature fostered hopes and generated surmises,
which he himself did not literally claim, and which have been to
a greater or less extent doomed to disappointment. That Koch
has inaugurated a method of great value for a limited number of
cases of tuberculosis (the superficial, the incipient and those that
can be supplimented by surgical measures) is undoubted, but for
another class of these cases its value seems to me extremely
apocryphal, and to expect from it an acquired and permanent immunity
would seem to contradict the nature of the disease.

				

